# Informing 21st-Century Risk Assessments with 21st-Century Science

**DOI:** 10.1289/ehp.1511135

**Published:** 2016-04-01

**Authors:** Linda S. Birnbaum, Thomas A. Burke, James J. Jones

**Affiliations:** 1 Office of the Director, National Institute of Environmental Health Sciences (NIEHS), National Institutes of Health (NIH), Department of Health and Human Services (DHHS), Research Triangle Park, North Carolina, USA; 2 Office of Research and Development, U.S. Environmental Protection Agency (EPA), Washington DC, USA; 3 Office of Chemical Safety and Pollution Prevention, U.S. EPA, Washington DC, USA

## Abstract

Understanding and preventing adverse impacts from chemicals in the environment is fundamental to protecting public health, and chemical risk assessments are used to inform public health decisions in the United States and around the world. Traditional chemical risk assessments focus on health effects of environmental contaminants on a chemical-by-chemical basis, largely based on data from animal models using exposures that are typically higher than those experienced by humans. Results from environmental epidemiology studies sometimes show effects that are not observed in animal studies at human exposure levels that are lower than those used in animal studies. In addition, new approaches such as Toxicology in the 21st Century (Tox21) and exposure forecasting (ExpoCast) are generating mechanistic data that provide broad coverage of chemical space, chemical mixtures, and potential associated health outcomes, along with improved exposure estimates. It is becoming clear that risk assessments in the future will need to use the full range of available mechanistic, animal, and human data to integrate multiple types of data and to consider nontraditional health outcomes and end points. This perspective was developed at the “Strengthening the Scientific Basis of Chemical Safety Assessments” workshop, which was cosponsored by the U.S. Environmental Protection Agency and the National Institute of Environmental Health Sciences, where gaps between the emerging science and traditional chemical risk assessments were explored, and approaches for bridging the gaps were considered.

## Introduction

Understanding and preventing adverse impacts from chemicals in the environment is fundamental to protecting public health, and scientifically sound chemical risk assessments are needed to support a variety of environmental protection decisions across the United States and around the world. Risk assessments provide qualitative information about a chemical’s health effects and quantitative information that helps inform the scope of national regulatory decisions, state and community decisions, and industry practices.

The 1983 four-step framework—hazard identification, dose response, exposure assessment, and risk characterization—developed by the National Research Council (NRC 1983) has shaped chemical risk assessments worldwide. However, the wide range of policy and regulatory applications within and across federal and state agencies in the United States and internationally, has led to an equally wide range of risk assessment practices. These different approaches may yield conflicting results and have contributed to concerns about the scientific credibility of risk assessments and related risk management decisions. The emergence of new methods in computational toxicology, exposure science, epidemiology, and systematic review hold great promise for advancing risk assessment. However, integration of these new approaches into established regulatory frameworks presents scientific and policy challenges.

The majority of regulatory frameworks guide risk assessment from the perspective of a single chemical or single component of a product formulation and often do not account for multiple chemical exposures and mixtures. Furthermore, most chemical risk assessments of potential human health effects rely on testing in animal models using exposures that are typically higher than those experienced by humans. This testing model requires the assessor to extrapolate to lower doses and across species, and it provides limited consideration of variability within species. All of these factors undermine confidence that current risk assessments are protective of human health, particularly for the most vulnerable individuals, communities, and life stages.

Results from environmental epidemiology studies have raised questions about whether traditional animal toxicology studies adequately predict health effects in human populations. These studies sometimes report effects that are not seen in animal studies, and hypothesis-based epidemiological studies may not yield data that can be easily incorporated into chemical risk assessments using existing frameworks and guidelines. Myriad publications in environmental and public health journals describe subtle chemical–biological interactions with population health effects that are not captured in traditional toxicity testing. The health effects observed in the epidemiological studies are typically different from end points evaluated in animal-based toxicity tests for hazard evaluation in chemical risk assessments. The real world exposure events depicted in epidemiology studies often do not correlate with exposures traditionally used in toxicity testing, which are most often much higher than exposures experienced in human populations. Furthermore, epidemiological studies incorporate background and chronic low-dose exposures that are not considered in traditional toxicity testing. Likewise, they may be able to capture population variability, which can be important for organizations charged with protecting public health.

Twenty-first century science is providing tremendous advances in systems biology, genomics and epigenetics, bioinformatics, exposure science, and environmental epidemiology, as well as innovations in chemical measurement and analytical technologies: All these advances are expanding our understanding of how chemicals can interact with biological systems. New approaches such as Toxicology in the 21st Century (Tox21; https://ncats.nih.gov/sites/default/files/factsheet-tox21.pdf) and exposure forecasting (ExpoCast; http://www.epa.gov/sites/production/files/2014-12/documents/exposure_forecasting_factsheet.pdf) are generating data that provide broad coverage of chemical space, chemical mixtures, and potential associated health outcomes, along with improved exposure estimates. Further development and use of systematic review will provide more transparency and more consistency and confidence in the integration of mechanistic, animal, and human data for use in risk assessments.

To provide a forum to discuss how science in the 21st century can bring about improvements in the risk assessment process, the U.S. Environmental Protection Agency (EPA) and the National Institute of Environmental Health Sciences (NIEHS) cosponsored the workshop “Strengthening the Scientific Basis for Chemical Safety Assessments,” which took place 15–16 July 2015 in Research Triangle Park, North Carolina. Participants included individuals with the diverse expertise in toxicology, epidemiology, and risk assessment needed to move this discussion forward. At the workshop, participants discussed the gaps in understanding between the new scientific methods and conventional approaches: These discussions led to proposed activities to bridge the gaps.

Introductory talks reviewed the growing evidence that exposures to a wide variety of chemicals encountered in daily life in the United States are linked to adverse health effects, including neurological deficits in children and adults, asthma, cardiovascular disease, and cancer. Invited speakers presented case studies to illustrate and provide background information for key topic areas including accounting for exposures during critical developmental windows, capturing variability in population susceptibility, translating experimental animal findings to humans, and addressing cumulative exposures. The group also discussed the perception prevalent in the public health community that chemical risk assessments, as currently carried out by the U.S. EPA and other agencies, are not sufficiently health protective. A strong theme among the participants was that new approaches have to be developed to incorporate data beyond traditional experimental and animal studies to support chemical safety evaluations that could prevent adverse health effects in the U.S. population.

## Understanding the Gaps between New and Conventional Methodologies

### The Chasm between Environmental Epidemiology and Risk Assessment

It was immediately apparent that there is limited understanding or familiarity, outside of the practitioners, with how risk assessments are conducted in federal regulatory agencies. As a consequence, most research investigations are not optimally designed to provide the types of information that are needed in current assessments. This lack of appropriate design can extend through the selection of study end points and the recording and reporting of findings, to an appreciation of required study design elements to inform a risk assessment. Conversely, current chemical risk assessment practices of the U.S. EPA and other federal agencies have not evolved to optimally consider and incorporate the information emerging from observational human research. Many risk assessment professionals lack understanding of epidemiology study designs, methods, strengths, and limitations. As a result, much potentially valuable information is excluded from the regulatory risk assessment process.

### Exposure, the Missing Link

Exposures, as observed, measured, and reported in environmental epidemiology studies, typically represent real-world exposures. These are often reported at levels below those used and delivered in traditional animal toxicology studies, and they are rarely confined to a single or few agents of concern, making it difficult to definitively elucidate causality in the hypothesized association with exposures. In contrast, when estimating the risks of use of a single pesticide, the assessment only considers the risk of exposure to the pesticide active ingredient, but the inert components in a pesticide formulation to which individuals are inevitably co-exposed, and which could modify the response, are usually not considered in the risk assessment. In addition, whereas exposures in observational studies encompass background exposures and chronic low-dose exposures to single or multiple chemicals, toxicological studies do not typically model or account for these.

### Importance of Life-Stage Exposures and Multigenerational Effects

Many of the environmental epidemiology studies discussed at the workshop probed the importance of windows of exposures, with the focus on parent and child exposures during critical life stages, such as preconception and perinatal periods, and understanding early-life determinants of lifelong diseases. While data from epidemiological studies also point to potential amplification of the adverse effects of these early-life exposures, including multigenerational effects, it was recognized that these effects are not modeled in standard toxicological studies or evaluated in risk assessments because data on which to judge such health outcomes are largely missing and not required. It will be necessary to expand approaches used to integrate mechanistic, animal, and human data in order to bridge gaps and inform risk assessment methods. The U.S. EPA and the NIEHS are committed to helping support cross-disciplinary efforts to achieve this goal.

### Understanding the Role of Nonchemical Stressors

There are many nonchemical stressors that are often overlooked in the conduct of risk assessments. Yet, current data, both from toxicological and epidemiological studies, demonstrate that physical agents such as light and noise, infectious agents, the microbiome, psychosocial factors, and nutrition can have significant impacts on health effects from chemical exposures. For example, while all the workshop participants agreed that stress is an important modifying factor for health, it is currently not considered as a risk cofactor or modeled in most studies on which risk assessments are based. However, the science that addresses biochemical markers of stress is evolving, and it was proposed that this evolution would be important to quantitatively study the interactions between stress and chemical exposures and account for both in chemical risk assessments. Stress is but one of many factors that may contribute to vulnerability within a population. Current practices that risk assessors use to account for vulnerability, such as uncertainty or safety factor adjustments, may not adequately capture true population vulnerability, such as that associated with stress or genetic variance. The extent to which this might be the case is currently unknown. There likely will be challenges in using data on nonchemical stressors in assessments that support regulatory action under various regulatory statutes in ways that promote improved public health.

### Influence of Funding Priorities

It was acknowledged that research specifically aimed at addressing a data gap for the purpose of a risk assessment is not likely to be funded through the typical grants review processes of the National Institutes of Health (NIH). Perhaps because of the funding priorities, academic investigators by and large study environmental exposures in the context of understanding their contributions to a disease or related adverse mechanistic event that is often not well aligned with the typical phenotypic end points measured and relied upon in regulatory or guideline toxicology studies. At this time, these types of toxicology studies are at the foundation of most chemical risk assessments that inform chemical safety evaluations. The U.S. EPA and the NIEHS recognize the value of fostering and funding such studies and collaborations. There is concern that the funding gap is likely to grow if not addressed systematically and deliberately.

## Proposed Bridging Activities

Based on discussions of immediate and longer-term activities to bridge the gaps in understanding, the workshop participants drew conclusions and recommended several activities.

### Increase Communication

It is important to find mechanisms to increase communication among researchers in multiple disciplines, including toxicology, epidemiology, and risk assessment. Suggestions were made to advance this communication. For example, the U.S. EPA could sponsor hands-on risk assessment experiences for researchers through short residential courses. Scientists within the U.S. EPA and outside the agency could also form scientific teams to work together to develop ways to improve the consideration and incorporation of epidemiology data into risk assessments, including in-depth analyses of study designs, dose metrics, confounding, and sampling issues. This could be accomplished by collaborating on consensus workshops or white papers.

### Enrich Funding Mechanisms

Teams of experimental and observational scientists and risk assessors could explore collaborations to design competitive grants programs that promote and fund studies to provide data of direct relevance to chemical risk assessment. This could be facilitated by the creation of dedicated grant review study sections.

### Examine Methods to Make Risk Assessments More Robust and Inclusive

It was suggested that teams carry out targeted case studies to examine how well risk assessment projections for “safe exposures” relate to exposures in a human population that are being linked to adverse health effects. To help with this examination, the U.S. EPA has asked the National Academies of Sciences, Engineering, and Medicine to help develop a strategy for evaluating whether the agency’s current regulatory toxicity-testing practices allow for adequate consideration of evidence of low-dose adverse human effects. A published report from this committee—Unraveling Low-Dose Toxicity: Case Studies of Systematic Review of Evidence (http://www8.nationalacademies.org/cp/projectview.aspx?key=49716)*—*is expected in early 2016 and could help improve understanding of cases where low-dose effects may have not been detected in current regulatory studies.

### Move Away from Reliance on Apical End Points

The risk assessment community should continue to explore ways to move away from the use of traditional phenotypic effects and outcomes in regulatory guideline animal safety assessment studies. Perturbations of molecular pathways involved in adverse phenotypic end points were considered to be potentially useful and might provide a better link between molecular epidemiology findings and traditional animal toxicology studies. It was suggested that the U.S. EPA and the NIEHS convene workshops to explore the relationships between *a*) observed potentially environmentally induced diseases, *b)* the typical phenotypic responses seen in animal toxicology studies, and *c*) the adverse outcome pathways (AOPs) that might relate the two together. A logical follow-on to these activities would be to expand studies to examine chemical interactions that work through different points of an AOP, particularly with respect to dose–response relationships and considerations of chemical mixtures.

Another suggestion was to simply begin to routinely use alternative end points, such as analyzing key characteristics of biologic pathways, for risk assessments, rather than only using apical health effects. Such analyses would require significant policy and possibly legislative changes, as well as significant outreach and education, considering some of the recent judicial interpretations of regulations promulgated under existing laws.

### Incorporate Interindividual Variability in Place of Default Safety Factors

One of the critical challenges in risk assessment is how interindividual variability and differential susceptibility are evaluated and incorporated. In order to address this challenge, scientific findings could be used in place of default uncertainty or safety factors to address population susceptibility. For example, one might use the profile of population variance in phase 1 or phase 2 enzyme activities for metabolism of selected chemical structures in place of default safety factors. It was pointed out, however, that variability in these enzymes in humans could be in excess of 100-fold. An alternate but related suggestion was to examine chemical structures agnostically with the intent of understanding chemical attributes that tend to produce highly variable responses across populations. This could be approached through an expansion of the Tox21–1000 Genomes Project (Abdo et al. 2015). Data from this project—with respect to 156 compounds in nearly 900 lymphoblastoid cell lines from five ethnic groups—has demonstrated that variability among individuals can be more than 200-fold. However, data from this project addresses genetic variability but does not consider other critical influences, such as life stage, diet, and the microbiome.

### Characterize and Incorporate Chemical Co-exposures

Methods need to be developed to incorporate emerging data on chemical co-exposures into risk assessments. Broader applications of novel technologies to examine patterns of common chemical co-exposures in populations, as well as advances in bioinformatics and in nontargeted chemical analyses of human biospecimens, hold promise to provide this in ways that avoid the current need for large volumes of blood (Guo et al. 2015). Current chemical risk assessments do not consider every stressor, since we do not have the data or know how to do this. Instead, they most often assess cumulative risk for common modes of action as required by law and supported by science. While imperfect, this may be a useful starting point for considering improving current practices and generating missing data on co-exposures.

### Study and Consider Multigenerational Effects

The potential significance of multigenerational inheritance of risks was noted, and there was recognition that this adds another vastly complicating dimension to understanding and estimating the risks of current exposures. This concept is one of the areas of scientific focus for the NIEHS 2012–2017 Strategic Goals (https://www.niehs.nih.gov/about/strategicplan/), and a systematic review of the current literature pertaining to this topic is underway. This comprehensive review will hopefully provide a basis for more definitive research in this area. Current multigenerational toxicity test methods in rats and other species have limitations, and there are opportunities to improve test guidelines and create integrated testing and assessment strategies that include mechanistic, animal, and human data.

### Relevance of Emerging Science to Risk Assessments

The U.S. EPA and NIEHS, in collaboration with other agencies, have asked the National Academies of Sciences, Engineering, and Medicine to provide guidance on integrating new scientific approaches into risk-based evaluations. The report from this committee—Incorporating 21st Century Science into Risk-Based Decisions (http://www8.nationalacademies.org/cp/projectview.aspx?key=49652)—is not expected to be published until early 2016, and as such, this topic was not central to the discussions at the meeting. Nevertheless, it was broadly acknowledged that there is currently little experience or precedence for incorporating the emerging 21st-century science into risk assessments: This includes the appropriate use of Tox21 high-throughput screening (HTS) information, commensurate high-throughput exposure estimations, nontargeted metabolomics, high-throughput transcriptomic, and other forms of emerging big data. This may also require an appreciation and understanding of how to assess the validity of proposed AOPs and networks and how to use them in informing risk assessments.

## Conclusions

Understanding the environmental determinants of disease and protecting susceptible and vulnerable populations are daunting scientific challenges. Addressing these challenges will require an inclusive multidisciplinary research approach and an improved recognition of the methods and informational needs of risk assessors. Progress will also require coordination across multiple federal programs and agencies—especially in resource-constrained times.

The 2015 workshop started with the recognition that there is a chasm between current risk assessment practices and evolving data from mechanistic and environmental epidemiological studies; it concluded with several concrete, practical, and achievable steps to help the U.S. EPA, the NIEHS, and the broader scientific community strengthen the scientific basis for chemical risk assessments. The resounding message of the workshop is that both federal agencies need to work with the research community to ensure that our assessments incorporate current science and consider the full range of vulnerabilities within the population. This research and assessement strategies are fundamental to our mission to ensure that our communities are safe, our air and water are clean, and our most vulnerable populations are adequately protected.
